# Phenotypic assortment in wild primate networks: implications for the dissemination of information

**DOI:** 10.1098/rsos.140444

**Published:** 2015-05-13

**Authors:** Alecia J. Carter, Alexander E. G. Lee, Harry H. Marshall, Miquel Torrents Ticó, Guy Cowlishaw

**Affiliations:** 1 Large Animal Research Group, Department of Zoology, University of Cambridge, Cambridge, UK; 2 The Institute of Zoology, Zoological Society of London, Regent's Park, London, UK; 3 Division of Ecology and Evolution, Department of Life Sciences, Imperial College London, Silwood Park, Berkshire, UK; 4 Centre for Ecology and Conservation, College of Life and Environmental Sciences, University of Exeter, Penryn, UK

**Keywords:** social network, social information, chacma baboon, personality, phenotypic assortment

## Abstract

Individuals' access to social information can depend on their social network. Homophily—a preference to associate with similar phenotypes—may cause assortment within social networks that could preclude information transfer from individuals who generate information to those who would benefit from acquiring it. Thus, understanding phenotypic assortment may lead to a greater understanding of the factors that could limit the transfer of information between individuals. We tested whether there was assortment in wild baboon (*Papio ursinus*) networks, using data collected from two troops over 6 years for six phenotypic traits—boldness, age, dominance rank, sex and the propensity to generate/exploit information—using two methods for defining a connection between individuals—time spent in proximity and grooming. Our analysis indicated that assortment was more common in grooming than proximity networks. In general, there was homophily for boldness, age, rank and the propensity to both generate and exploit information, but heterophily for sex. However, there was considerable variability both between troops and years. The patterns of homophily we observed for these phenotypes may impede information transfer between them. However, the inconsistency in the strength of assortment between troops and years suggests that the limitations to information flow may be quite variable.

## 2. Introduction

Individuals can acquire information either personally, by interacting with their environment directly, or socially, by attending to the behaviour of others [1]. For group-living animals, social information provides an alternative to gathering costly personal information [2], potentially facilitating decisions about foraging, movement, mate choice and predation [3,4]. Social information allows the rapid dissemination of novel information among group members and has thus also been widely implicated in the formation of traditions and cultures within species [5,6].

An individual's phenotype is likely to influence its propensity to either ‘generate’ social information—through personal information acquisition—or ‘exploit’ it—from information ‘generators’. In the first case, for instance, certain phenotypes are more likely to solve cognitive tasks or discover novel foods than others. Sex, age, previous experience [7] and personality [8,9] can affect an individual's propensity to solve tasks. In the second case, personality phenotypes can affect the acquisition (and use) of social information. Shy geese (*Branta leucopsis*) [10] and baboons (*Papio ursinus*) [11] are more likely to use social information than their bolder conspecifics while foraging. Thus, the diffusion of novel information through a group may not show a uniform pattern of transmission, but rather a more heterogeneous pattern in which some individuals are generating information while others are exploiting it, either directly from generators or secondarily from other exploiters.

The dissemination of information through a group, however, is also predicted to differ with the social network—the patterning of social relationships—that characterizes that group [12,13]: individuals are predicted to be more likely to acquire social information from others with whom they associate more frequently [12]. Assortment by phenotype has often been observed in animal social networks [14–16]. A preference to associate with similar phenotypes, termed positive assortment or ‘homophily’, is thought to be adaptive by potentially facilitating cooperation among individuals [14–17]. However, homophily, combined with phenotype-specific information generating, may preclude some individuals from obtaining social information. This is because the individuals with phenotypes that generate information may be more likely to associate with others of similar phenotype and not with those who might rely more heavily on social information. On the other hand, negative assortment (‘heterophily’) may facilitate the transfer of information between information generators and exploiters. Thus, the propagation of information through a social network should be limited or enhanced by positive or negative assortment of information-generating phenotypes, respectively.

Researchers have tested whether information will transfer between individuals who are in close proximity and have assumed that measures of proximity reflect opportunities for information transmission between individuals (e.g. [18–20]). However, we still know very little about which behavioural measures of association best indicate information transmission opportunities, nor whether the type of behavioural measure matters for the complexity of information that has to be transferred. Indeed, animal social networks can be described using several different measures of association [21], and these are not always comparable [22,23]. This has important implications for understanding information flow in a network. For instance, individuals may be more attentive towards, and thus more likely to acquire or use information from, those with whom they have strong interactive bonds rather than those with whom they simply share space [12]. This supposition is supported by Boogert *et al.* [24], who found that starlings (*Sturnus vulgaris*) preferentially learnt novel foraging techniques from others with whom they perched, rather than from individuals with whom they foraged in close proximity. Importantly, these may not necessarily be the same individuals in both networks [22–25]. Thus, to understand the potential for information dissemination through social networks, there is a need to consider more than one method of measuring associations between individuals in those networks.

In this study, we investigated whether social networks of wild baboons were assorted by phenotype to explicitly address for the first time to our knowledge the potential for the dissemination of social information. We chose to study chacma baboons for several reasons. First, chacma baboons generate and exploit information according to phenotype: juveniles and bolder baboons are more likely to solve novel foraging tasks, thus generating novel information [9], while higher ranked, shyer individuals are more likely to acquire social information when foraging with unreliable cues [11]. Second, baboons show highly differentiated, non-random social relationships [22,23] and all individuals within a baboon troop are identifiable and travel together as a group, alleviating the issues of missing individuals that occurs in most other social network studies [21]. Third, baboons learn socially about novel foods from others [9,26], and it is reasonable to predict that individuals will learn from others with whom they are affiliated [12]. Finally, we have a uniquely rich dataset describing the social networks and phenotypes of over 100 baboons in two troops over a 6 year period. We quantified social networks using two measures of behavioural association between individuals to ask whether baboon troops were assorted by individual phenotype. We considered four phenotypic traits that may influence the propensity to generate/exploit social information, namely sex, dominance rank, age and boldness. We also assigned individuals two further traits that directly estimated an individual's propensity to generate and exploit social information, respectively, on the basis of combinations of these four basic traits. To our knowledge, this is the first study to explore assortativity with such broad scope; the majority of studies describing social network characteristics occur either over one study period [16], in one study group [14,15], or use one method of determining a connection [14,16], limiting the generalizability of their findings for understanding the fine structure of animal social networks and the potential for the diffusion of social information. Within our broader question, and because of the scope of our data and analyses, we were able to further describe whether the patterns we observed could be generalized: (i) between the observation groups, (ii) between the measures of behavioural association, and (iii) among the years of observation.

## 3. Material and methods

### 3.1 Study site and species

We studied chacma baboons over 6 years, from May to November 2009, May to October 2010, June to September 2011, August to October 2012, August to October 2013 and May to July 2014 at Tsaobis Leopard Park, Namibia (15°45′ E, 22°23′ S). Two troops of baboons (J-troop and L-troop, study individuals present: *n*_2009_=J=35, L=24; *n*_2010_=32, 23; *n*_2011_=27, 23; *n*_2012_=31, 22; *n*_2013_=46, 49; *n*_2014_=44, 48) have been habituated to the presence of observers at close range and are individually recognizable. We collected data annually from adult, sub-adult and juvenile baboons over the 2009–2014 period. Age (in years) was estimated from a combination of known birth dates and dental patterns of tooth eruption and wear [27]. Unmarked immigrant males' ages were estimated at 9 years old when they appeared in the study troops, as this is the age most males were observed to transfer from our study groups. Individual ranks were assessed through dominance interactions that were recorded during focal observations and ad libitum using Matman v. 1.1.4 (Noldus Information Technology 2003). Dominance hierarchies in both troops were strongly linear in all years (Landau's corrected linearity index: *h*′>0.6, *p*<0.05 in both troops in all years). Dominance rank was expressed relatively (which controls for group size), calculated from absolute ranks for each baboon using the formula 1−[(1−*r*)/(1−*n*)], where *r* is the individual's absolute rank and *n* is the group size of the individual. Individual rank values therefore range from 0 (lowest rank) to 1 (highest rank) in each group year.

Boldness was measured in 2009, 2010, 2011, 2013 and 2014 by scoring responses to a novel food (for further details, see [28,29]). In all cases, individuals were presented with a stimulus when they were alone and moving between food patches. The stimuli were presented on the edges of game trails and paths regularly used by the baboons. All experiments were filmed to facilitate data extraction (Panasonic SDR-SW20, Kadoma Osaka, Japan; see movie files in [29]). Stimuli for the boldness tests consisted of novel food items which included hard-boiled eggs with the shell on or removed, or a small egg-shaped bread roll, all of which were dyed red or green (Moir's food dye), in 2009; semi-dried eighths of apple or pear, dyed red, in 2010; and eighths of an orange or equivalent-sized pieces of butternut squash in 2011; 3×2 cm pieces of carrot or gemsquash in 2013; and 2×3 cm pieces of potato or sweet potato dyed blue in 2014. Any naive individual that saw another individual interacting with a novel food was presented with a different novel food when they were tested. As an indication of an individual's willingness to interact with novelty and thus its boldness, we recorded the amount of time spent inspecting the food item (s) between approaching the food item and the end of the test, which was determined to be when the baboon either left or ate the item. If an individual did not inspect the novel food they scored 0, while inspection time for those individuals who inspected the food had a cut-off value of 120 s. In total, 58, 54, 50, 95 and 92 baboons received novel food presentations in 2009, 2010, 2011, 2013 and 2014, respectively (median number of presentations per individual=2.0; range=1–5 presentations). The substantial increase in sample size from 2013 onwards reflects the inclusion of a new generation of juvenile animals. Owing to funding limitations, boldness tests were not completed in 2012.

Previous research in this population indicates that the probability of generating or exploiting social information is not necessarily related to single traits but a combination of traits [11,29]. In light of this, we also categorized individuals according to: (i) their propensity to generate information, if they were bolder than average (median) for a given year's boldness scores for the troop *and* juvenile (younger than 5 years for females, 8 for males) for that year [9]; and (ii) their propensity to exploit information, if they were shyer than average (median) *and* higher ranking than average (relative rank greater than 0.5) for that year [11]. We have analysed these separately (as information generator/not and information exploiter/not), as some individuals do not necessarily fall into an information generator/exploiter phenotype; for example, low ranking but bold adults are neither an information generator nor exploiter by our previous findings. Furthermore, in J troop in 2011, there were no individuals categorized as information exploiters because all higher ranking individuals inspected the novel food for longer than the median inspection time. In 2012, we could not estimate individuals' likely propensities to generate or exploit information as we did not measure boldness. While this is a crude categorization, it could still usefully illuminate patterns of assortment in likely information generators and exploiters that are potentially missed by analysing the constituent phenotypes (boldness, age and rank) separately.

### 3.2 Behavioural data collection

Observers followed the baboon troops from dawn until dusk during the periods of study. The behaviour of individual baboons was recorded using continuous focal sampling for periods of 15–60 min during the full-day troop follows. Focal observations that lasted under 15 min were discarded. Individuals were sampled in a semi-random manner such that the cumulative focal observation time for each individual was even in each of four time periods over each day (06.00–09.00, 09.00–12.00, 12.00–15.00 and 15.00–18.00). If an individual disappeared from the troop for greater than half the field season (either owing to emigration or death; *n*_2009_=3, *n*_2010_=3, *n*_2011_=1, *n*_2012_=2, *n*_2013_=1 and *n*_2014_=0), the individual was removed from the analyses for that year. Between 2009 and 2012, we avoided sampling females while they were in oestrus due to known changes in their social interactions with other troop members at this time [30,31]. They were, however, recorded when they were in close proximity to or observed grooming with other individuals under observation. Because of time and funding constraints in 2013 and 2014, we included oestrus females as focal individuals and collected the network data slightly differently (see below). Thus, only data from 2009 to 2012 are directly comparable among years; however, we have also presented the 2013 and 2014 data for within-year comparisons between the troops.

We recorded data using two methods to define an association. First, as information could transfer between individuals who are in close proximity (e.g. [20]), we continuously recorded the nearest neighbour [32] within 5 m of the focal individual during focal observations. If the focal baboon had no neighbour within 5 m, it was recorded as ‘alone’. Assuming that an individual is more likely to acquire information from another the more time they spend together, we calculated the proportion of each individual's total observation time that it spent in close proximity to every other individual in the group. In 2013, owing to a change in protocol that made continuous recording of the nearest neighbour inaccurate, we instead recorded the nearest neighbour every 2 min during focal follows. Thus, in 2013, we calculated the proportion of each individual's total nearest neighbour scans that it spent in close proximity to every other individual in the group. In 2014, due to funding limitations that limited the numbers of observers in the field, the nearest neighbour data were recorded as scans independently of focal follows. In these scans, we searched for and quantified the nearest neighbour (within 5 m) of ‘focal’ individuals from a randomized list of study individuals within each troop. Because the baboon troops can spread over 1 km^2^ while foraging (A. J. Carter 2009–2014, personal observations) and finding particular individuals can be time consuming, the observer (M.T.T.) searched for one of the first five individuals on the randomized list of baboons to optimize the number of independent nearest neighbour dyads that could be sampled each day. If a focal baboon had already been recorded in an already-sampled subgroup (i.e. subgroup membership had not changed between scans), that individual was not sampled for an hour to ensure that the sampled subgroups constituted independent data. We calculated the total number of times each individual was observed as the nearest neighbour of every other individual in the group.

Second, as information may preferentially be acquired by individuals who have strong social bonds [12], we recorded the time each focal individual spent grooming every other individual in the group. Grooming is an easily defined and observable social interaction and is also a standard measure of affiliative associations in primates. We calculated the proportion of each individual's total grooming time that it spent grooming every other individual in the group. Although grooming associations are not strictly independent from nearest neighbour associations (the nearest neighbour of a grooming baboon is usually the grooming recipient), we have previously found that networks created using these different association measures were generally not correlated [23]. We chose to normalize grooming for each individual instead of calculating the proportion of focal observation time dedicated to grooming each individual, as we felt that it better reflected particular *individuals*' strategic investment in their relationships with others. Owing to funding limitations in 2014, grooming data were collected ad libitum as events when observers moved through the troops. For each event, we recorded the identities and direction of the grooming dyads. To avoid pseudoreplication, and because we could not know who initiated every grooming event, an independent grooming bout was recorded only when the partner identities of a grooming dyad changed, or grooming ceased and the dyad moved to a different location. As such, we did not record reversals of dyads within one grooming bout, i.e. we did not record a new grooming record when individual A was observed grooming individual B, and B then groomed A without moving to a new location.

### 3.3 Statistical analyses

We created association matrices that were both weighted, as the proportion of time spent in proximity and grooming could vary between 0 and 1, and directed, as in both cases the connection could not be mutually returned. This resulted in a total of 24 association matrices for the combinations of troops, years and association methods. We then tested whether each network was assorted by rank (ranging from 0 to 1), sex (male/female), boldness (the natural log of (the time spent inspecting the novel food +1)), age (1–26 years), and propensity to generate information (high/low) and exploit information (high/low) using the *assortnet* package [33] in the statistical software R v. 3.0.3 [34]. Weighted assortativity calculates the degree to which associations occur between individuals of similar phenotypes and ranges from −1, where individuals are always found with individuals of a different phenotype, to 1, where individuals are always found with individuals of the same phenotype [33]. In the case of sex and the propensity to generate/exploit information, we calculated discrete assortment, and in all other cases we calculated continuous assortment. Standard errors, calculated by jackknife simulation [33], that do not overlap 0 indicate significant assortativity in the network structure.

In our analysis, we assessed the effects of each phenotype independently rather than concurrently in a single test, because there is no statistical technique that will investigate the effects of multiple phenotypes on (weighted) network assortativity. However, one implication of this approach is that it may lead to spurious relationships if the phenotypes are intercorrelated. To assess whether this was the case, we created correlation matrices of the four uncombined phenotypes for the troops and years (see the electronic supplementary material, table 1). We focused on pairwise correlations approaching or exceeding |*r*|>0.70 as a critical threshold beyond which collinearity may be a serious problem [35]. Of the possible 66 combinations of phenotypic correlations across troops and years, only 4 (6%) approached or exceeded this threshold. These reflected positive correlations between sex and rank (adult males are higher ranking than females) in both troops in 2011 and 2012 (when the proportion of adults in the sample was at its highest; the relationship between sex and rank is much weaker in juveniles). However, these correlations did not appear to lead to any spurious relationships in our analysis, as the observed patterns of assortment by sex and rank were in the opposite rather than the same direction (see below).

**Table 1.** Assortativity (± s.e.) for social networks based on nearest neighbour proximity (nn) and grooming (groom) associations for four phenotypic traits over the years 2009–2014 for two troops of baboons (J, L). (n.a. refers to phenotypes which were not measured in that particular year. Dashes (—) refer to phenotypes that did not occur in a particular year. Statistically significant assortment is indicated in italics.)

			boldness	relative rank	age (years)	sex	propensity to generate information	propensity to exploit information
year	troop	network	assortativity index	assortativity index	assortativity index	assortativity index	assortativity index	assortativity index
2009	J	nn	−0.024±0.035	*0.130*±*0.044*	*0.143*±*0.062*	*0.076*±*0.045*	*0.104*±*0.046*	0.004±0.053
2009	L	nn	*0.074*±*0.053*	*0.282*±*0.048*	*0.069*±*0.060*	0.012±0.056	*0.099*±*0.061*	0.040±0.061
2010	J	nn	0.022±0.069	*0.107*±*0.061*	0.038±0.108	0.032±0.055	*0.065*±*0.060*	0.023±0.068
2010	L	nn	*0.110*±*0.107*	*0.248*±*0.087*	−0.058±0.099	−0.007±0.065	*0.064*±*0.050*	*0.154*±*0.148*
2011	J	nn	0.028±0.054	*0.103*±*0.054*	0.070±0.070	−0.025±0.059	*0.075*±*0.054*	—
2011	L	nn	0.004±0.070	0.016±0.057	−0.027±0.065	−*0.161*±*0.050*	−*0.060*±*0.031*	0.060±0.104
2012	J	nn	n.a.	−0.052±0.060	0.043±0.057	−*0.095*±*0.051*	n.a.	n.a.
2012	L	nn	n.a.	0.015±0.069	0.008±0.056	−*0.217*±*0.044*	n.a.	n.a.
2013	J	nn	*0.055*±*0.024*	*0.256*±*0.025*	*0.068*±*0.030*	*0.049*±*0.029*	*0.107*±*0.029*	*0.075*±*0.028*
2013	L	nn	*0.078*±*0.052*	*0.151*±*0.035*	*0.058*±*0.030*	−*0.073*±*0.039*	*0.116*±*0.031*	0.010±0.056
2014	J	nn	*0.116*±*0.027*	*0.065*±*0.037*	*0.176*±*0.032*	−0.025±0.029	*0.158*±*0.027*	*0.077*±*0.025*
2014	L	nn	*0.094*±*0.026*	0.026±0.033	*0.213*±*0.016*	−*0.125*±*0.024*	*0.134*±*0.021*	0.006±0.036
2009	J	groom	*0.216*±*0.018*	*0.188*±*0.037*	*0.176*±*0.056*	*0.263*±*0.048*	*0.220*±*0.051*	−*0.073*±*0.051*
2009	L	groom	−0.050±0.081	*0.560*±*0.053*	*0.113*±*0.108*	−0.019±0.060	*0.120*±*0.067*	−0.036±0.093
2010	J	groom	−0.023±0.076	−*0.039*±*0.008*	*0.132*±*0.083*	−*0.131*±*0.022*	−*0.136*±*0.034*	*0.256*±*0.003*
2010	L	groom	*0.230*±*0.105*	*0.313*±*0.053*	−*0.156*±*0.070*	−*0.132*±*0.054*	−*0.061*±*0.005*	*0.240*±*0.127*
2011	J	groom	*0.115*±*0.015*	−*0.143*±*0.017*	*0.023*±*0.003*	−*0.158*±*0.033*	*0.139*±*0.009*	—
2011	L	groom	−*0.231*±*0.023*	*0.120*±*0.026*	−0.010±0.046	−*0.052*±*0.038*	−*0.053*±*0.036*	−*0.094*±*0.004*
2012	J	groom	n.a.	*0.095*±*0.019*	*0.162*±*0.024*	−*0.066*±*0.036*	n.a.	n.a.
2012	L	groom	n.a.	0.082±0.131	*0.107*±*0.056*	−*0.332*±*0.044*	n.a.	n.a.
2013	J	groom	*0.067*±*0.003*	*0.044*±*0.020*	−*0.196*±*0.003*	−*0.154*±*0.017*	*0.200*±*0.028*	*0.055*±*0.009*
2013	L	groom	−*0.076* ±<*0.001*	*0.359* ±<*0.001*	−*0.094*±<*0.001*	−*0.288*±<*0.001*	*0.169*±<*0.001*	*0.142*±*0.000*
2014	J	groom	−0.012±0.033	−*0.069*±*0.060*	*0.076*±*0.032*	−*0.277*±*0.027*	0.021±0.032	0.031±0.048
2014	L	groom	*0.165*±*0.041*	−0.016±0.021	*0.129*±*0.025*	−*0.474*±*0.022*	*0.118*±*0.024*	−0.021±0.022

## 4. Results

**Figure 1.**
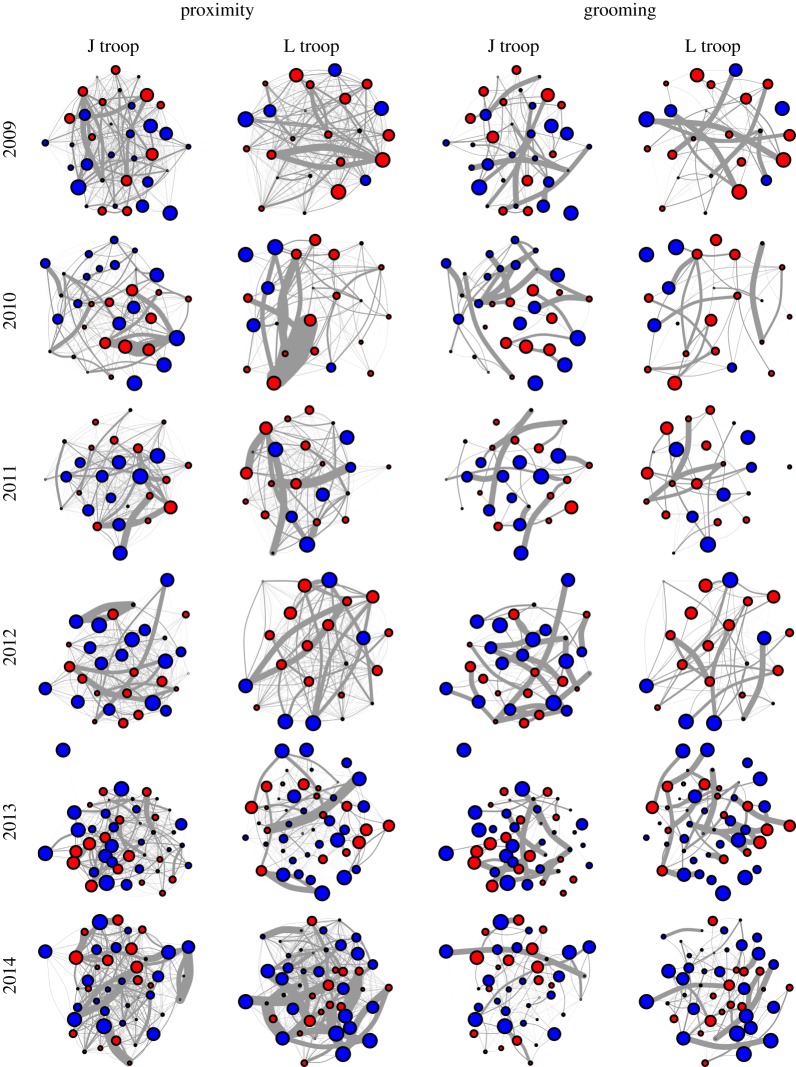
Social networks based on the time spent in close proximity to (left) and grooming (right) other individuals, for two troops of baboons (J and L) over 6 years (2009–2014). Each node indicates an individual, and the lines connecting the nodes represent the connections between individuals. Node colour indicates sex (blue, male; red, female), while node size indicates rank (larger nodes designate higher ranks). Line thickness indicates the connection strength, with thicker lines designating stronger connections (i.e. a greater proportion of time spent in close proximity to or grooming). However, note that the line thickness is not comparable between proximity and grooming (we have increased line thickness in the proximity networks to make weaker connections more obvious). Node position is conserved within years for each troop.

**Figure 2.**
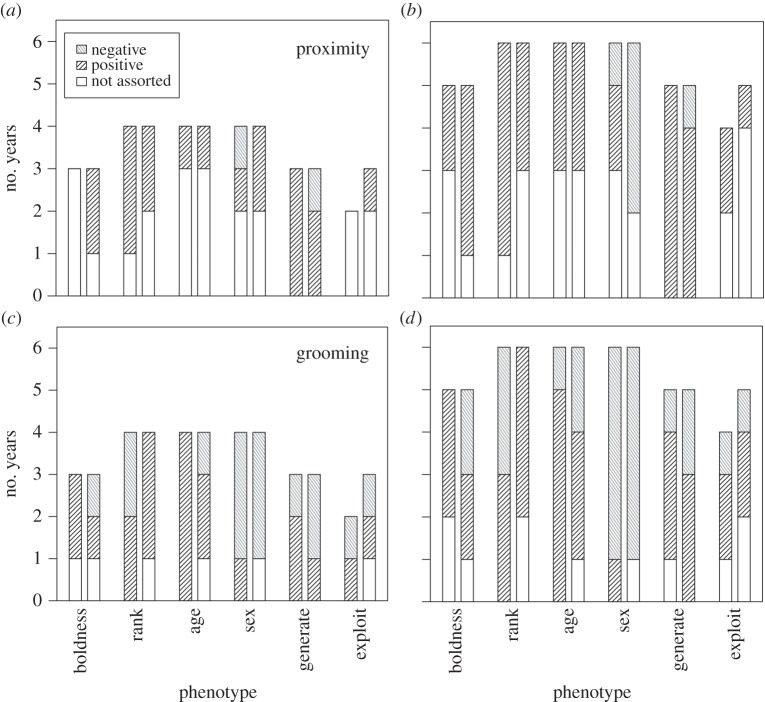
Network assortment patterns for six phenotypes in two troops of baboons. Counts of the types of assortment over 4 years (*a*,*c*) and 6 years (*b*,*d*) are presented for proximity (*a*,*b*) and grooming (*c*,*d*) networks. Assortment type is indicated by shading (see legend). Each phenotype shows the assortment for J (left bars) and L (right bars) troops.

In total, we created 24 social networks based on two methods of measuring a connection between individuals for the two troops in the six study years (figure 1). We then tested whether these networks were assorted by the four phenotypes (table 1 and figure 2). In the four years with comparably collected data (2009–2012), we found that grooming networks were almost twice as likely to be assorted as proximity networks (35 out of 41 versus 19 out of 41 networks, or 85% versus 46%, respectively) (table 1). Information generators were assorted in every grooming network (100%, or 6 out of 6 cases). Age, sex and rank all led to equally high levels of assortment in the grooming networks (88%, or 7 out of 8 networks, in each case), followed by boldness (67%, 4 out of 6) and the propensity to exploit information (1 out of 5, 20%). Phenotypic assortment in the proximity networks was not only lower but much more variable, being most common in relation to the propensity to generate and exploit information (100%, 6 out of 6; and 80%, 4 out of 5; respectively), followed by rank (63%, 5 out of 8), sex (50%, 4 out of 8), boldness (33%, 2 out of 6) and age (25%, 2 out of 8). Phenotypic assortment was also high for all traits in both network types in both 2013 (96%, 23 out of 24 cases) and 2014 (66%, 16 out of 24 cases).

In grooming networks, where phenotypic assortment occurred, the patterns were consistently positive for rank, age and boldness (71%, 5 out of 7; 86%, 6 out of 7; 75%, 3 out of 4), equal for the propensity to generate or exploit information (50%; 3 out of 6; 50%, 2 out of 4) but negative for sex (88%, 7 out of 8). Thus, animals of similar rank, age and boldness, but different sex, were more likely to be associated when grooming; by contrast, no such associations were observed according to the propensity to generate/exploit information. Similar patterns were observed in the proximity networks in those cases where assortment occurred. Similar patterns were also observed in 2013 and 2014 in both network types, with the exception that those with a propensity to generate/exploit information were more likely to be positively assorted in both years and age-assortativity reversed in the 2013 grooming networks.

In general, the groups also showed similar patterns of phenotypic assortment (figure 2). Looking within years at each phenotype–network combination, when significant assortment was observed in one group it was also usually observed in the other. However, there were a substantial minority of instances (34%, 17 out of 50) where this was not the case. Similarly, where both groups showed significant assortment, the direction of assortment was usually the same, but there were a number of occasions when the direction differed (24%, 8 out of 33). These differences were most common for boldness (50%, 2 out of 4), followed by propensity to generate information (33%, 2 out of 6), rank (29%, 2 out of 7), sex (14%, 1 out of 7), age (13%, 1 out of 8) and propensity to exploit information (0%, 0 out of 2).

## 5. Discussion

We assessed, for the first time to our knowledge, broad-scale patterns of individual assortativity in animal social networks in relation to the potential generation and exploitation of social information. Using baboons as a model system, we tested whether individuals were assorted by six phenotypic traits which can influence an individual's mode of social information use: sex, rank, age, boldness and the propensity to generate (bolder juveniles) and exploit (higher ranking shyer individuals) information phenotypes. We demonstrated that: (i) assortment was common in grooming networks, which overall showed homophily for rank, age, boldness and the propensity to generate and exploit information, but heterophily for sex; (ii) proximity networks were less frequently assorted, in approximately half of the cases, but similar and more consistent patterns of homophily and heterophily among years were observed where assortment did occur; and (iii) between the two troops, there were broadly similar patterns in the occurrence and direction of assortment, albeit with considerable variability. Our results have implications for understanding the transfer of information between individuals in social networks, and we discuss these implications below before considering the importance of broader investigations in animal social network studies.

As a rule, if only animals of a particular phenotype are information generators, then any degree of homophily in that phenotype is going to impede information transmission to other group members. We have previously reported that younger (juvenile) and bolder baboons are more likely to solve novel foraging tasks [9]. This suggests that these individuals have a propensity to generate information and may therefore often act as information generators in the troop (although all individuals have the potential to act as information generators at some point). If so, our finding that grooming networks typically showed homophily in both these phenotypes, and the combination of these phenotypes (propensity to generate information), indicates that the diffusion of information through baboon troops may be restricted by its social structure. It remains to be seen whether the lower instances of assortment in many proximity networks is sufficient to compensate for these effects, though—importantly for our hypothesis—the vast majority of proximity networks showed homophily for those with a propensity to generate information. Heterophily, which would actively promote information transmission, was only consistently found in one phenotype, namely sex, which was previously found to be unrelated to solving novel foraging tasks [9]. As we mention in the Introduction, naive individuals may acquire social information directly from information generators or secondarily from information exploiters. The limitations to information transmission due to homophily that we describe may apply only to those cases of direct transmission between information generators and exploiters; however, after an initial primary transfer between an information generator to an exploiter, homophily may then promote information transmission among secondary information exploiters.

We see our ‘information phenotype’ framework as an exciting future research direction. We encourage researchers that are interested in understanding information transmission processes to integrate individual differences in information phenotypes with the social environment. Our previous work investigating individual differences in information use [9,11] and that of others which investigate the consistency of foraging strategies that rely on the use of personal and social information [10,36] suggest that individuals may differ consistently in how they collect and process information. This will have implications for the transmission of information in groups (we develop this further below). However, the categorization of individuals according to their propensity to generate/exploit information, while usefully based on combinations of other phenotypes here, requires further exploration. While we are not suggesting that individuals will exclusively generate or exploit information, we predict that individuals will have a tendency to prefer one information acquisition strategy over the other. This preference may be based on their phenotype, as we have suggested, and/or their position in their social network and thus their opportunities to exploit information. Future research could also investigate whether individual differences in information plasticity (i.e. the flexibility with which individuals switch between generating and exploiting information) could also be predicted by an individuals' phenotype [36]. For example, some individuals in our study could be categorized as neither high nor low propensity to generate/exploit information; it is possible that these individuals are more flexible in their use of information acquisition strategies.

Our goal in this study was to predict whether information transfer would be promoted or retarded by patterns in the fine structure of social networks, specifically through the assortment of phenotypes that may influence modes of information acquisition. However, one aspect of social information transfer that may limit our ability to predict its transmission through social networks involves the difference between social information acquisition and its use. We have shown evidence that some individuals may acquire social information but not use it ([9], see also [37]), which would limit how far information could diffuse through a network. It may be unreasonable to assume that an individual will always use the social information that it has acquired [37], but this possibility is rarely, if ever, considered in studies modelling social information diffusion (e.g. [38]). Individual differences in the use of acquired social information have two far-reaching implications for studies not only of social information diffusion but also for studies of social learning, and traditions and culture in animal societies. First, simulation studies of social information diffusion and social learning probably consistently overestimate the rates at which information can diffuse among individuals in a network. This is because simulations assume that information will always diffuse between naive and informed individuals even though informed individuals may not demonstrate their knowledge [9]. Such knowledgeable non-demonstrators should be considered functionally naive in simulations, as they cannot or will not demonstrate to naive individuals, even if they associate at high rates. Second, because social information will not transfer from informed but non-demonstrative individuals to truly naive individuals, the formation of traditions in animal societies may not necessarily be limited by individuals' abilities to acquire a novel skill, but by individual differences in the propensity to use that skill once it has been acquired. Simulations and descriptive studies like ours may be useful for exploring the opportunities for information transfer between individuals and the particular inter-individual routes this information might take. However, direct measurement of information flow is needed in different social networks through time in order to establish whether it actually occurs.

We considered two types of network for possible information transmission. These were based on grooming and proximity associations. Although we cannot currently say which is most important for information transmission, it seems likely that both will play a role. Assuming that information transmission requires close visual contact [9], spatial proximity will be important, though we emphasize that this will depend on the type and complexity of the information that is being generated. However, among their neighbours, individuals may be most attentive to those with whom they share strong social (grooming) bonds (cf. [12,24]). The finding that grooming networks showed stronger assortment than proximity networks suggests that assortment may largely reflect patterns of social affiliation, but that these assortment patterns are attenuated in proximity networks as the signal is diluted (or in some cases reversed) by the movements of animals which take them away from the immediate vicinity of preferred social partners. Previous research in baboons has shown that social bonds are often formed with animals of similar age and rank, at least among adult females, who form the stable core of the group (*Papio cynocephalus*: [39], *P. ursinus*: [40]). The findings of homophily in these assortment patterns are therefore expected. The finding of heterophily in the assortment patterns for sex is more surprising, as female baboons tend to prefer forming bonds with other females [41], but most likely reflects the combined effects of male–male avoidance and the prevalence of female–male grooming interactions during both oestrus (between sexual partners) [42] and lactation (between mothers and male ‘friends’ who protect their infants) [42].

No previous research has considered whether bolder/shyer baboons are more likely to associate with each other or not; our analysis is, to our knowledge, the first to suggest that this can happen, and although the pattern is variable, it is in line with previous findings in chimpanzees (*Pan troglodytes*) [15] and guppies (*Poecilia reticulata*) [14]. These previous studies have suggested that homophily for personality traits could promote cooperation among individuals. In guppies, for example, boldness is measured by a tendency to inspect a predator [14], and cooperative predator inspection occurs more frequently among affiliated individuals [17], thus homophily for boldness may facilitate cooperation among bolder associates. Why baboons should demonstrate homophily for boldness is unclear, unless boldness has a heritable component (e.g. [44]), and these patterns reflect family associations.

While the diffusion of information in baboon groups may often be limited by homophily, we also observed substantial variation in assortment both between troops and years (see also [23]). One possible methodological source of inter-annual variation within the 2009–2012 period is the diminishing proportion of juvenile animals sampled. Between 2009 and 2012, while the numbers and identities of individuals sampled in each troop remained relatively constant, the proportion of the troop that was sampled decreased as the numbers of infants and juveniles increased with the births that occurred after 2008 (the last occurrence of individual marking). In 2013 and 2014, the sample size of individuals increased again, as did the proportions of the members of study troops that were included in the networks, following troop capture and a new round of individual marking in 2012 for all those animals born since 2008 (figure 1). It is therefore possible that the similar patterns of assortment observed in 2009 and 2013 partially reflect the similar proportional sampling of the troops (all weaned baboons) in these 2 years. Nevertheless, inter-annual variation in the biological processes that we have discussed above and go on to describe below is also very likely to contribute to these patterns. This methodological complexity highlights two important considerations, both for our current study and more generally. First, the role of juveniles in contributing to the fine structure of a network may be overlooked, especially for long-lived, social mammals such as many primates and cetaceans, where relationships with and between juveniles are often ignored (e.g. [39,40]). In our study population, juveniles are particularly important in their role as information generators [9], and omitting them from social networks may generate different predictions and give different results from those networks in which they are included. Second, continual demographic changes in networks make direct comparisons between time periods difficult, especially in the wild where demographic changes cannot be controlled ([23], cf. [45]). Thus, researchers studying social networks in the wild will often have to make decisions about which individuals to include and how to follow them longitudinally—either: (i) the same core group of individuals is followed and their relationships with individuals that immigrate, emigrate, are born and die are largely ignored; or (ii) all individuals are included and confounds between demographic and temporal changes are accepted as a complication of longitudinal studies. We deal further with the issue of demographic changes below.

Changes in group demography, and the associated alterations in social networks, are also very likely to contribute to the variation in assortment between troops and among years [45–47]. For example, the immigration of several non-natal adult males in both troops between 2011 and 2014 may have contributed to the stronger patterns of negative assortment by sex in their proximity networks in those years. Furthermore, extreme inter-annual environmental variation at the study site (A. J. Carter 2009–2014, personal observation) may have caused changes in social structure, as has been found in other baboon populations in response to changes in season [48]. As mentioned earlier, male–female relationships will be affected by females' reproductive states, and it is likely that stochastic variation in the proportion of females in any one state at any one time might affect the network structure and patterns of assortment within it. Furthermore, female–female relationships will be affected by female reproductive state in at least two ways: (i) mothers with infants attract more grooming than at times when they do not have an infant [49,50], and (ii) oestrus females are the targets of aggression from other females [30], which presumably will affect both proximity and grooming interactions among females. The finding of extreme inter-annual variation has two implications. First, while the transfer of information may be limited by the social network at some points in time, individuals are likely to have access to information generators at others. Thus, the flow of information may be promoted or limited by temporal variation in the patterning of relationships. Second, high temporal variability in networks makes it difficult to generalize about the flow of information in social networks from a single point in time; a full understanding of how information diffuses through social networks is likely to require assessments at multiple time periods or in multiple groups.

## Supplementary Material

Supplementary Table 1: Correlation matrices of the phenotypes for two troops of baboons over 6 years.

## Data accessibility

Networks and phenotype data are available on figshare. Figshare (http://dx.doi.org/10.6084/m9.figshare.1239171).
