# Supplementary material for: Phenotypic assortment in wild primate networks: implications for the dissemination of information
Source: R Soc Open Sci. 2015 May 13;2(5):140444. doi: 10.1098/rsos.140444 (PMC4453262; doi:10.1098/rsos.140444)
Supplement: Supplementary Table 1: Correlation matrices of the phenotypes for two troops of baboons over 6 years. [file rsos140444supp1.docx]

**Phenotypic assortment in wild primate networks: implications for the dissemination of information**

Alecia J. Carter, Alexander E. G. Lee, Harry H. Marshall, Miquel Torrents Ticó & Guy Cowlishaw

Supplementary Table 1: Correlation matrices of the phenotypes for two troops of baboons over 6 years.

| **Year** | **Troop** | **Phenotype** | **Boldness** | **Rank** | **Age** | **Sex^a^** |
| --- | --- | --- | --- | --- | --- | --- |
| 2009 | J | Boldness | 1 |  |  |  |
|  |  | Rank | -0.07 | 1 |  |  |
|  |  | Age | -0.16 | 0.29 | 1 |  |
|  |  | Sex | 0.36 | 0.23 | -0.38 | 1 |
|  | L | Boldness | 1 |  |  |  |
|  |  | Rank | -0.11 | 1 |  |  |
|  |  | Age | -0.46 | 0.22 | 1 |  |
|  |  | Sex | 0.35 | 0.22 | -0.33 | 1 |
| 2010 | J | Boldness | 1 |  |  |  |
|  |  | Rank | -0.03 | 1 |  |  |
|  |  | Age | 0.04 | -0.16 | 1 |  |
|  |  | Sex | 0.24 | 0.49 | -0.43 | 1 |
|  | L | Boldness | 1 |  |  |  |
|  |  | Rank | 0.04 | 1 |  |  |
|  |  | Age | -0.48 | -0.04 | 1 |  |
|  |  | Sex | 0.35 | 0.45 | -0.3 | 1 |
| 2011 | J | Boldness | 1 |  |  |  |
|  |  | Rank | 0.33 | 1 |  |  |
|  |  | Age | -0.32 | -0.33 | 1 |  |
|  |  | Sex | 0.57 | 0.78 | -0.45 | 1 |
|  | L | Boldness | 1 |  |  |  |
|  |  | Rank | 0.23 | 1 |  |  |
|  |  | Age | -0.39 | 0.02 | 1 |  |
|  |  | Sex | 0.47 | 0.69 | -0.27 | 1 |
| 2012 | J | Boldness |  |  |  |  |
|  |  | Rank |  | 1 |  |  |
|  |  | Age |  | -0.37 | 1 |  |
|  |  | Sex |  | 0.78 | -0.46 | 1 |
|  | L | Boldness |  |  |  |  |
|  |  | Rank |  | 1 |  |  |
|  |  | Age |  | -0.21 | 1 |  |
|  |  | Sex |  | 0.63 | -0.26 | 1 |
| 2013 | J | Boldness | 1 |  |  |  |
|  |  | Rank | 0.06 | 1 |  |  |
|  |  | Age | -0.36 | 0.09 | 1 |  |
|  |  | Sex | 0.4 | 0.43 | -0.43 | 1 |
|  | L | Boldness | 1 |  |  |  |
|  |  | Rank | -0.2 | 1 |  |  |
|  |  | Age | -0.43 | 0.24 | 1 |  |
|  |  | Sex | 0.35 | 0.31 | -0.43 | 1 |
| 2014 | J | Boldness | 1 |  |  |  |
|  |  | Rank | -0.16 | 1 |  |  |
|  |  | Age | -0.48 | -0.04 | 1 |  |
|  |  | Sex | 0.37 | 0.43 | -0.42 | 1 |
|  | L | Boldness | 1 |  |  |  |
|  |  | Rank | -0.22 | 1 |  |  |
|  |  | Age | -0.42 | 0.21 | 1 |  |
|  |  | Sex | 0.13 | 0.42 | -0.43 | 1 |

Shaded cells highlight strong correlations (|*r*| > 0.70) among the phenotypes.

**^a^** Sex was coded as an integer: females = 0, male = 1
